# A pilot study on the validity and psychometric properties of the electronic EQ-5D-5L in routine clinical practice

**DOI:** 10.1186/s12955-021-01898-3

**Published:** 2021-12-18

**Authors:** Cindy Lo Kuen Lam, Emily Tsui Yee Tse, Carlos King Ho Wong, Joyce Sau Mei Lam, Sikky Shiqi Chen, Laura Elizabeth Bedford, Jason Pui Yin Cheung, Calvin Kalun Or, Paul Kind

**Affiliations:** 1 Department of Family Medicine, The University of Hong Kong Shenzhen Hospital, Shenzhen, China; 2 Department of Family Medicine and Primary Care, Li Ka Shing Faculty of Medicine, The University of Hong Kong, 3/F, Ap Lei Chau Clinic, 161 Main Street, Ap Lei Chau, Hong Kong, SAR China; 3 Department of Pharmacology and Pharmacy, Li Ka Shing Faculty of Medicine, The University of Hong Kong, Hong Kong, SAR China; 4 Department of Orthopaedics and Traumatology, Li Ka Shing Faculty of Medicine, The University of Hong Kong, Hong Kong, SAR China; 5 Industrial and Manufacturing Systems Engineering, Faculty of Engineering, The University of Hong Kong, Hong Kong, SAR China; 6 Department of Applied Health Research, Institute of Epidemiology and Health Care, University College London, London, UK

**Keywords:** Health-related quality of life, Psychometrics, Musculoskeletal problem, Electronic EQ-5D-5L, Clinical practice

## Abstract

**Background:**

Electronic measurement of health-related quality of life (HRQOL) may facilitate timely and regular assessments in routine clinical practice. This study evaluated the validity and psychometric properties of an electronic version of the EQ-5D-5L (e-EQ-5D-5L) in Chinese patients with chronic knee and/or back problems.

**Methods:**

151 Chinese subjects completed an electronic version of the Chinese (Hong Kong) EQ-5D-5L when they attended a primary care or orthopedics specialist out-patient clinic in Hong Kong. They also completed the Chinese Western Ontario and McMaster University Osteoarthritis Index (WOMAC), a Pain Rating Scale, and a structured questionnaire on socio-demographics, co-morbidities and health service utilization. 32 subjects repeated the e-EQ-5D-5L two weeks after the baseline. 102 subjects completed e-EQ-5D-5L and 99 completed the Global Rating on Change Scale at three-month clinic follow up. Construct validity was assessed by the association of EQ-5D-5L scores with external criterion of WOMAC scores. We tested mean differences of WOMAC scores between adjacent response levels of the EQ-5D-5L dimensions by one-way ANOVA, test–retest reliability by intra-class correlation, sensitivity by known group comparisons and responsiveness by changes in EQ-5D-5L scores over 3 months.

**Results:**

There was an association between EQ-5D-5L and WOMAC scores. Mean WOMAC scores increased with the increase in adjacent response levels of EQ-5D-5L dimensions. Test–retest intraclass correlation coefficient (ICC) of EQ-5D-5L utility and EQ-VAS scores were 0.76 and 0.83, respectively, indicating good reliability. There were significant differences in the proportions reporting limitations in the EQ-5D-5L dimensions, the utility and VAS scores between the mild and severe pain groups (utility = 0.28, *p* = 0.001; VAS = 11.46, *p* < 0.001), and between primary care and specialist out-patient clinic patients (utility = 0.15, *p* = 0.001; VAS = 10.21, *p* < 0.001), supporting sensitivity. Among those reporting ‘better’ global health at three-months, their EQ-5D-5L utility and EQ-VAS scores were significantly increased from baseline (utility = 0.18, *p* < 0.001; VAS = 10.75, *p* = 0.005).

**Conclusions:**

The electronic version of the EQ-5D-5L is valid, reliable, sensitive and responsive in the measurement of HRQOL in Chinese patients with chronic knee or back pain in routine clinical practice.

**Supplementary Information:**

The online version contains supplementary material available at 10.1186/s12955-021-01898-3.

## Background

Health-related quality of life (HRQOL) is the assessment of aspects of quality of life influenced by an individual's health [1]. HRQOL can be used as an outcome measure to assess the impact of illnesses and the effect of interventions on patients, monitor the health conditions of individual patients, and evaluation of quality of care [2–4]. The EQ-5D is a widely used HRQOL measure. It was first developed in Europe and later adapted to many other languages and cultures including Chinese in mainland China and Hong Kong [5–8]. It has been shown to be valid, reliable and responsive in general populations and specific patient groups in different cultures around the world [9–13].

The EQ-5D includes five items where respondents self-report any problems in relation to mobility, self-care, daily activities, pain/discomfort, and anxiety/depression. The original version, the EQ-5D three-level (EQ-5D-3L), contains three response options: ‘no problems’, ‘some/moderate problems’ and ‘extreme problems/unable to’ for each of the five items [14, 15]. In 2011, the EQ-5D-3L was updated to the EQ-5D five level (EQ-5D-5L), which increased the response options from three to five (‘no problems’, ‘slight problems’, ‘moderate problems’, ‘severe problems’, ‘extreme problems/unable to’) to enhance sensitivity [16, 17]. The EQ-5D has been shown to be a useful HRQOL measure in providing a more holistic picture of the health of patients [11, 18], for monitoring responses to treatment/surgery [19, 20], assessing the quality of care [21] and for health-economic evaluation [19]. In addition, the completion of HRQOL measures has been found to enable patients to be more aware of their health conditions and how the diseases affect them, which empower them to raise any issues or concerns with their clinicians [22, 23].

There have been increasing attempts to incorporate HRQOL measures in routine clinical care [18–20], however many barriers have been encountered, including high workload of staff [3, 4, 23] and a lack of time to collect, analyze and interpret the data [24]. Furthermore, some clinicians have questioned the validity and sensitivity of HRQOL data and are concerned that implementing HRQOL assessment may disrupt patient care [3] and increase patient burden [23]. Given these barriers, there have been calls for ways in which HRQOL data can be more effectively integrated into routine clinical practice [4, 25]. One such method is through electronic data collection and reporting [4]. Aside from the benefit of reducing workload and time burden on staff, it can also allow clinician’s immediate access to the results and tracking of changes [3, 18].

An electronic version of the EQ-5D-5L (e-EQ-5D-5L) has been available since 2014 [26] and many studies have applied e-EQ-5D-5L to measure HRQOL outcomes [27–30]. Although there is a large body of literature supporting the validity and psychometric properties of paper versions of EQ-5D [12, 13, 31–33], there are few such data on e-EQ-5D-5L. Our literature search found one recent study in English and French asthma patients reporting the validity and psychometric properties of e-EQ-5D-5L [34] but no study in the Chinese population. A change in the mode of administration can affect the validity, reliability and other psychometric properties of an instrument. Electronic administration can be challenging for many older Chinese patients in Hong Kong who have low education levels and are not familiar with computer technology. It is essential to confirm the validity and psychometric properties of e-EQ-5D-5L before it can be applied to clinical practice in Hong Kong and other Chinese populations especially in settings that have large elderly patient populations.

This pilot study aimed to test the validity and psychometric properties of e-EQ-5D-5L as a measurement of the HRQOL of patients with chronic knee and/or back problems in routine clinical practice. The objectives were to evaluate the construct validity, test–retest reliability, sensitivity and responsiveness of e-EQ-5D-5L among Chinese patients with chronic knee and/or back problems attending outpatient clinics in Hong Kong.

## Methods

### Study design, subject recruitment and data collection

This was a prospective longitudinal cohort study. We recruited patients with chronic knee and/or back problems by convenience sampling when they attended a public primary care general out-patient clinic (GOPC) and a public orthopedics specialist out-patient clinic (SOPC) in Hong Kong between August and November of 2018. Eligible patients were invited by either their doctors or trained research assistants to join the study. These public outpatient clinics were busy with an average workload of 6 to 8 patients per hour per doctor, therefore we could not invite all eligible patients who attended the clinics during the study period due to manpower constraints. The subject inclusion criteria were: 1) adults aged 18 years or above; 2) a doctor-diagnosed symptomatic chronic (≥ one month) knee and/or back problem; 3) attending the clinic for a doctor consultation and was scheduled for a follow-up visit to the clinic within 12 months; 4) able to communicate in Chinese; and 5) able to provide written consent to participate. Patients whose life expectancies were estimated to be less than 3 months by the attending doctors, or those who were too ill (either physically or cognitively) to complete the questionnaires, or those who were not willing or unable to give consent were excluded.

All subjects completed a written informed consent before participating in the study. Each subject was assigned a unique QR code for access to the e-EQ-5D-5L survey, and completed the electronic version of the Chinese (Hong Kong) EQ-5D-5L and EQ-VAS (e-EQ-5D-5L) online through an iPad that was connected to a central server via the clinic public Wi-Fi. One item was presented per screen and the subject could choose to move to the next item after completion or to skip the item. The original 200 mm EQ-VAS was modified to 100 mm to fit into the iPad screen. The detailed administration method of the e-EQ-5D-5L with screenshots is shown in the Additional file 1: Appendix 1. Trained research assistants (RA) were present on site to provide technical assistance and to read out the questions to respondents as required by some elderly subjects who had low literacy level or poor eyesight. Immediately after the subject had completed the e-EQ-5D-5L, the RA retrieved the report summarizing the EQ-5D dimension, utility and VAS scores from the server and printed a copy of the report for the consulting doctor’s information. Most subjects completed the e-EQ-5D-5L survey before seeing their doctors so that they could show the reports to their doctors during the consultations. A few subjects who were recruited by the doctors completed the survey after the doctor consultation. In addition to the e-EQ-5D-5L, subjects completed the paper-based WOMAC, Pain Rating Scale and a structured questionnaire on socio-demographics, co-morbidities (self-reported doctor-diagnosed chronic diseases with a duration of ≥ one month) and health services utilization.

We invited the first 51 subjects recruited from the GOPC to return to the clinic two weeks after their baseline visit to repeat the e-EQ-5D-5L to evaluate test–retest reliability. All subjects (including the first 51 subjects) who attended the clinics for follow up around 3 months post-baseline were asked to complete the e-EQ-5D-5L and Global Rating Scale on change in health (GRS). We took 3 months as the interval for reassessment as it is standard practice in the GOPC that patients with stable chronic problems are followed up every 3 months. On the other hand, the SOPC follow up interval would usually be longer for stable cases.

Research ethics approval was obtained from the institutional review board prior to subject recruitment. (HKU/Hospital Authority Hong Kong West IRB reference number: UW 18–270).

### Study instruments

#### The Chinese (Hong Kong) EQ-5D-5L

The EQ-5D-5L comprises five items representing five HRQOL dimensions (mobility, self-care, usual activities, pain/discomfort and anxiety/depression) and a Visual Analogue Scale (EQ-VAS) on global health. The responses to the five EQ-5D-5L items have a combination of 3125 (5^5^) health states [5, 16]. Each health state can be converted to a composite utility (preference) score from 0 (death) to 1 (perfect health), with a scoring algorithm derived from population-based valuation. The Chinese-Traditional (Hong Kong) translation of the EQ-5D-5L and the Hong Kong population specific EQ-5D-5L value set have been developed and normed on the local population [6, 8]. The EuroQol Group full version of EQ-5D-5L (Web version)—Chinese-Traditional (Hong Kong) was adapted for electronic administration. The EQ-VAS was modified from the original 200 mm to a 100 mm scale from 0 (the worst imaginable health state) to 100 (the best imaginable health state), in order to fit into the iPad screen.

#### Additional PROMs administered

- The *Western Ontario and McMaster University Osteoarthritis Index (WOMAC)* is a widely used condition-specific HRQOL measure to assess pain, stiffness and difficulty in physical functioning among patients with musculoskeletal conditions. It has been administered to patients with hip and/or knee osteoarthritis [35] and low back pain [36]. It consists of 24 items in 3 domains: pain (5 items), stiffness (2 items) and physical function (17 items). Each item is rated on a 5-point Likert scale, ranging from 0 to 4, with higher scores indicating more symptoms or greater impairment. The item scores in each domain are summated as the domain score. The total WOMAC score is the sum of the three domain scores [37]. A Chinese version of WOMAC is available and has been shown to be valid, reliable and sensitive in Chinese patients [37, 38].
- The *Pain Rating Scale* was administered to assess the severity of pain, scores range from 0 (no pain) to 10 (the worst pain).
- *The Global Rating Scale on change in health (GRS)* was used to assess the patient’s perception of any change in their overall health condition on a 7-point scale, ranging from much worse [1] to much better [7] at their 3-month follow up [39].

### Statistical analysis

Data were analyzed using IBM SPSS version 26. Statistical significance was set at a p value of < 0.05. Construct validity of the e-EQ-5D-5L was assessed by its association with the external criterion of WOMAC, based on the hypothesis that subjects with a higher level of problem/impairment in the EQ-5D-5L dimensions would have higher WOMAC domain and total scores if the e-EQ-5D-5L is a valid measure of HRQOL. To assess correlations between e-EQ-5D-5L and WOMAC, one-way analysis of variance (ANOVA) along with post-hoc least significant difference was applied to compare the mean differences of WOMAC scores across levels of EQ-5D-5L dimensions, and between adjacent response levels (level 1 vs 2 vs 3 +).

Test–retest reliability of the e-EQ-5D-5L utility and EQ-VAS scores was assessed by intra-class correlation (ICC). A standard of ≥ 0.7 signifies good reliability [40]. Mean differences in EQ-5D-5L utility and EQ-VAS scores between baseline and 2-week re-test were also assessed by paired t tests. Test–retest reliability of the EQ-5D-5L dimension levels was assessed by examining the Gwet’s agreement coefficients (AC) and degree of agreement for five individual EQ-5D-5L dimension responses. A Gwet’s AC and degree of agreement of < 0.2 was interpreted as poor reliability between two assessments, 0.21–0.4 as fair, 0.41–0.6 as moderate, 0.61–0.8 as good and ≥ 0.8 as very good[41].

Sensitivity was measured by the ability of the e-EQ-5D-5L to detect a difference between groups (mild pain versus severe pain groups, GOPC versus SOPC groups, and knee pain versus back pain groups), tested by two-sample t tests. We also assessed the magnitude of the difference by Cohen's effect size [42], calculated as the difference between mean scores, divided by pooled standard deviations (SD).

We conducted trajectory analyses fitting censored normal mixture models to determine the changes in EQ-5D-5L utility and EQ-VAS scores at baseline and 3 months after baseline, and disaggregated subjects into trajectory classes. The best model with up to five classes was selected using the Bayesian Information Criteria (BIC) [43].

For assessing responsiveness, we hypothesized that participants with “better” Global Rating Scale scores would have an increase in the EQ-5D-5L utility and EQ-VAS scores and that those with “worsened” GRS scores would have reductions in these EQ-5D-5L scores. We categorized the responses of 1 (much worse), 2 (worse) and 3 (a little worse) as the “worsened”; 4 (No change) as the “same”; and 5 (a little better), 6 (better) and 7 (much better) as the “better” groups, respectively. Mean changes in EQ-5D-5L utility and EQ-VAS scores measured during follow-up visits at the clinics around 3 months after baseline in subjects with GRS ‘worsened’, ‘same’ and ‘better’ health were calculated and evaluated by paired t-tests and Cohen’s effect size. Chi squared tests were used to compare the difference in changes in the proportions of reported limitations in the EQ-5D-5L dimensions among the better, same and worsen groups in GRS.

## Results

### Subject characteristics

**Fig. 1:**
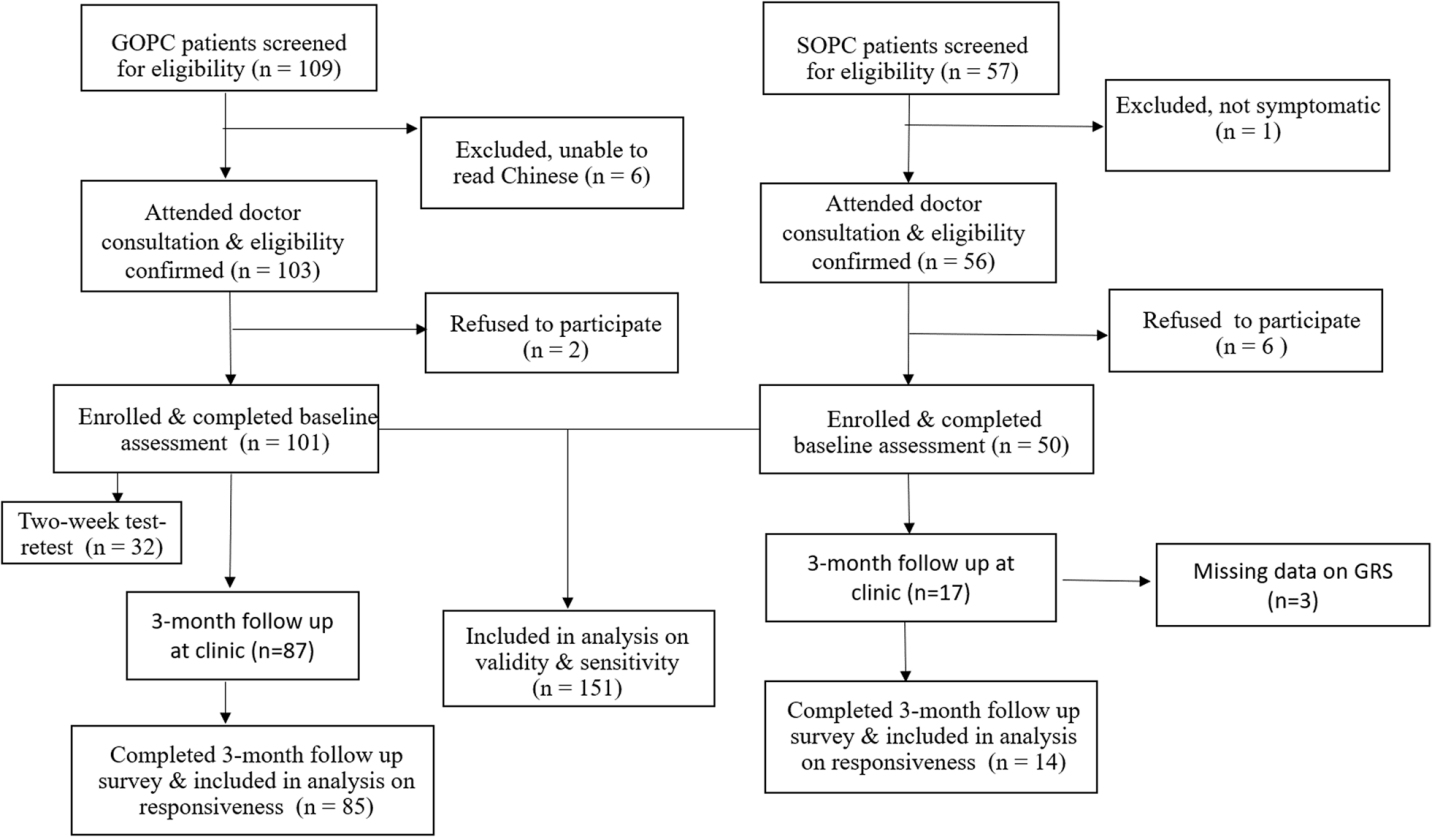
Study flow diagram

A total of 151 adult subjects with chronic knee and/or back problem (101 from GOPC and 50 from SOPC) participated in this study. Thirty-two subjects from the GOPC repeated the electronic EQ-5D-5L two weeks post-baseline. 104 subjects had attended follow-up consultations around 3 months at the clinics, while 47 subjects (14 GOPC patients, 33 SOPC patients) did not because they were not due for follow up or they defaulted their appointments. We missed the follow up of two GOPC subjects who attended follow up consultations during the weekends when our research assistants were off duty. 102 subjects (85 from GOPC and 17 from SOPC) completed the three-month follow-up assessment, but 3 subjects from the SOPC group did not respond to the GRS. Hence data from only 99 subjects were included in the analysis on responsiveness. The subject recruitment and follow-up flow diagram is shown in Fig. 1.

Baseline socio-demographic and clinical characteristics of the subjects are presented in Table 1. Overall, the subjects were mostly older adults (mean age: 64.8 years ± 9.23, range 36 to 89 years old), and many (45%) had low education levels of primary school or less. In terms of diagnoses, 35% had chronic back problems, 58% had chronic knee problems, and 6.6% of subjects had both. Subjects from the SOPC were relatively younger (mean age: 61.5 years ± 9.73) with the female gender predominant (74%) when compared to subjects from the GOPC. SOPC patients had a higher mean WOMAC and pain rating scores indicating more severe diseases than those of GOPC patients. The number and percentage of subjects reporting at different levels of the EQ-5D-5L at baseline are also presented in Table 1.

**Table 1:** Baseline characteristics of study subjects in 2018 (N = 151)

	Overall (N = 151)	GOPC (N = 101)	SOPC (N = 50)	*P* value†
*Socio-Demographics (%, n)*				
Male	53.0 (80)	66.3 (67)	26.0 (13)	0.001
Female	47.0 (71)	33.7 (34)	74.0 (37)	
Age (mean ± SD), year	64.77 ± 9.23 (151)	66.13 ± 8.42 (101)	61.52 ± 9.73 (50)	0.001
Education				0.154
Primary or less	45.0 (68)	52.5 (53)	30.0 (15)	
Secondary	47.0 (71)	40.6 (41)	60.0 (30)	
Tertiary	7.9 (12)	6.9 (7)	10.0 (5)	
*Diagnosis (%, n)*				< 0.001
Back	35.1 (53)	16.8 (17)	72.0 (36)	
Knee	58.3 (88)	78.2 (79)	18.0 (9)	
Both	6.6 (10)	5.0 (5)	10.0 (5)	
Duration of Diagnosis				0.001
< 1 year	15.2 (23)	17.8 (18)	10.0 (5)	
1–< 5 years	45.0 (68)	32.7 (33)	70.0 (35)	
5–10 years	17.9 (27)	17.8 (18)	18.0 (9)	
> 10 years	21.9 (33)	31.7 (32)	2.0 (1)	
Number of other Chronic Diseases				0.399
0	14.6 (22)	10.9 (11)	22.0 (11)	
1–3	72.8 (110)	76.2 (77)	66.0 (33)	
> = 4	12.6 (19)	12.9 (13)	12.0 (6)	
*WOMAC (mean ± SD, n)*				
WOMAC Total score	27.69 ± 17.09 (149)	23.98 ± 16.07 (100)	35.92 ± 17.22 (50)	0.001
WOMAC Pain Subscale score	6.87 ± 4.06 (151)	6.11 ± 3.99 (101)	8.40 ± 3.81 (50)	0.001
WOMAC Stiffness Subscale score	2.48 ± 2.00 (150)	1.95 ± 1.84 (100)	3.54 ± 1.90 (50)	0.001
WOMAC Function Subscale score	18.45 ± 12.36 (150)	15.68 ± 11.18 (100)	23.98 ± 12.84 (50)	0.001
*Pain Rating Scale scores (out of 10)*	4.37 ± 2.35 (151)	3.94 ± 2.45 (101)	5.24 ± 1.90 (50)	0.001

EQ-5D-5L Dimensions Level % (n)				*P* value^§^
Mobility (% with Limitation)#	28.4	24.7	36.0	0.074
No problems	25.2 (38)	26.7 (27)	22.0 (11)	
Slight problems	46.4 (70)	48.5 (49)	42.0 (21)	
Moderate problems	20.5 (31)	17.8 (18)	26.0 (13)	
Severe problems	7.9 (12)	6.9 (7)	10.0 (5)	
Extreme problems/unable to	0 (0)	0 (0)	0 (0)	
Self-care (% with Limitation)#	7.3	6.9	8.0	0.403
No problems	72.8 (110)	77.2 (78)	64.0 (32)	
Slight problems	19.9 (30)	15.8 (16)	28.0 (14)	
Moderate problems	6.6 (10)	6.9 (7)	6.0 (3)	
Severe problems	0.7 (1)	0 (0)	2.0 (1)	
Extreme problems/unable to	0 (0)	0 (0)	0 (0)	
Usual activities (% with Limitation)#	21.9	10.9	44.0	< 0.001
No problems	41.7 (63)	50.5 (51)	24.0 (12)	
Slight problems	36.4 (55)	38.6 (39)	32.0 (16)	
Moderate problems	17.2 (26)	6.9 (7)	38.0 (19)	
Severe problems	4.0 (6)	4.0 (4)	4.0 (2)	
Extreme problems/unable to	0.7 (1)	0 (0)	2.0 (1)	
Pain (% with Limitation)#	47.7	40.6	62.0	0.007
No problems	9.3 (14)	13.9 (14)	0 (0)	
Slight problems	43.0 (65)	45.5 (46)	38.0 (19)	
Moderate problems	35.1 (53)	30.7 (31)	44.0 (22)	
Severe problems	11.3 (17)	8.9 (9)	16.0 (8)	
Extreme problems/unable to	1.3 (2)	1.0 (1)	2.0 (1)	
Anxiety/depression (% with Limitation)#	15.9	9.9	28.0	0.002
No problems	51.0 (77)	60.4 (61)	32.0 (16)	
Slight problems	33.1 (50)	29.7 (30)	40.0 (20)	
Moderate problems	11.3 (17)	7.9 (8)	18.0 (9)	
Severe problems	3.3 (5)	2.0 (2)	6.0 (3)	
Extreme problems/unable to	1.3 (2)	0 (0)	4.0 (2)	
EQ-5D-5L Utility score (mean ± S.D.)	0.62 ± 0.26	0.67 ± 0.23	0.52 ± 0.27	0.001
EQ-VAS score (mean ± S.D.)	67.89 ± 17.09	71.3 ± 15.78	61.06 ± 17.75	< 0.001

GOPC = general outpatient clinic; SOPC = specialist outpatient clinic; WOMAC = Western Ontario and McMaster University Osteoarthritis Index

^#^EQ-5D-5L dimension responses of moderate, severe and very severe were grouped into the “with Limitation” category

^**†**^Tested by independent t-test; § tested by Chi-square test

### Validity and reliability

All subjects completed the e-EQ-5D-5L with no missing data at baseline. The mean completion time was 129.9 s (SD: 59.3; range 40 to 402 s). As presented in Table 2, the sign of mean differences in WOMAC scores between each EQ-5D-5L adjacent response levels were in the same direction. The differences in WOMAC scores between adjacent response levels of the EQ-5D dimensions were significant except the mean WOMAC Stiffness scores between level 1 and 2 of Mobility, level 2 and 3+ of Self-Care, level 2 and 3+ of Usual Activities and level 1 and 2 of Pain/Discomfort. There were significant correlations between the total WOMAC score and the EQ-5D-5L utility (-0.628) and EQ-VAS (-0.485) scores. We explored whether educational level had an effect on the e-EQ-5D-5L results, and found no statistically significant difference in the EQ-5D-5L, utility and EQ-VAS scores among three education level (primary or less, secondary and tertiary) groups.

**Table 2:** Comparison of WOMAC scores among EQ-5D-5L response levels at baseline in 2018 (N = 151)

EQ-5D dimension	Adjacent response level (n)	WOMAC Score							
		Pain		Stiffness		Function		Total	
		mean diff	*p* value	mean diff	*p* value	mean diff	*p* value	mean diff	*p* value
Mobility			< 0.001†		0.022†		< 0.001†		< 0.001†
	1–2 (38, 70)	− 2.238	0.003*	− 0.100	0.801*	− 7.403	0.001*	− 9.948	0.002*
	2–3+ (70, 43)	− 2.617	< 0.001*	− 0.954	0.014*	− 7.181	0.001*	− 10.855	< 0.001*
Self-Care			< 0.001†		0.036†		< 0.001†		< 0.001†
	1–2 (110, 30)	− 1.936	0.014*	− 0.804	0.050*	− 7.107	0.002*	− 10.033	0.002*
	2–3+ (30, 11)	− 3.818	0.005*	− 0.421	0.545*	− 12.879	0.001*	− 17.118	0.002*
Usual Activities			< 0.001†		< 0.001†		< 0.001†		< 0.001†
	1–2 (63, 55)	− 2.404	< 0.001*	− 1.439	< 0.001*	− 10.056	< 0.001*	− 14.010	< 0.001*
	2–3+ (55, 33)	− 3.152	< 0.001*	− 0.552	0.174*	− 8.939	< 0.001*	− 12.721	< 0.001*
Pain/Discomfort			< 0.001†		< 0.001†		< 0.001†		< 0.001†
	1–2 (14, 65)	− 1.623	0.112*	− 0.453	0.398*	− 5.682	< 0.089*	− 7.758	0.083*
	2–3+ (65, 72)	− 3.974	< 0.001*	− 1.656	< 0.001*	− 8.922	< 0.001*	− 14.485	< 0.001*
Anxiety/Depression			< 0.001†		< 0.001†		< 0.001†		< 0.001†
	1–2 (77, 50)	− 1.841	0.007*	− 0.907	0.006*	− 5.510	0.005*	− 8.289	0.002*
	2–3+ (50, 24)	− 2.807	0.003*	− 1.702	< 0.001*	− 12.365	< 0.001*	− 16.983	< 0.001*

3+: responses 3 to 5

*p value of pairwise multiple comparison adjusted by least significant difference;

^†^One-way ANOVA were applied to compare among response levels in five dimensions of EQ-5D scores

Table 3 showed the test–retest results of the Intraclass Correlation Coefficient (ICC) of EQ-5D-5L utility and EQ-VAS scores being 0.76 and 0.83 respectively, signifying good reliability. The proportions of level of agreement for each EQ-5D-5L dimension response between baseline and the 2-week re-test were as follows: 59% (mobility), 72% (self-care), 59% (usual activities), 59% (pain/discomfort), and 66% (depression/anxiety), indicating moderate to good reliability.

**Table 3:** Test–retest reliability of the electronic EQ-5D-5L in subjects from GOPC 2018 (N = 32)

	Baseline (N = 32)			2-week Follow-up (N = 32)			Gwet's AC	Agreement, %
	% with limitation	Floor (%)	Ceiling (%)	% with limitation	Floor (%)	Ceiling (%)		
Mobility	21.9	0.0	18.8	25.1	0.0	25.0	0.475	59.0
Self-care	9.4	0.0	68.8	3.1	0.0	75.0	0.669	72.0
Usual activities	12.5	0.0	50.0	15.6	0.0	40.6	0.513	59.0
Pain/discomfort	53.2	0.0	6.3	37.5	0.0	9.4	0.514	59.0
Anxiety/depression	15.6	0.0	50.0	6.3	0.0	56.3	0.557	66.0

	Mean (SD)	Floor (%)	Ceiling (%)	Mean (SD)	Floor (%)	Ceiling (%)	*p* value†	ICC
EQ-5D Utility Mean (SD)	0.66 (0.19)	0.0	7.8	0.67 (0.23)	N.A	9.4	0.718	0.757
EQ-VAS	72.19 (16.36)	0.0	3.9	74.53 (15.73)	0.0	6.3	0.282	0.834

SD = standard deviation; ICC = Intraclass correlation; N.A. = Not Applicable; Gwet's AC = Gwet's agreement coefficient

^†^Tested by paired t-test; EQ-5D-5L dimension responses of moderate, severe and very severe were grouped into the ‘with limitation’ category; Ceiling for EQ-5D-5L score = 1; Floor for EQ-5D-5L score = 5

### Sensitivity

Table 4 shows the sensitivity of the e-EQ-5D-5L dimensions, utility and EQ-VAS in detecting a difference between different known groups. The mild pain group had significantly lower proportions of subjects reporting limitation (moderate, severe and very severe responses) in all five EQ-5D dimensions and significantly higher utility scores and EQ-VAS scores, compared to the severe pain group. When compared to subjects from the SOPC, subjects from the GOPC had significantly lower proportions of limitation in the EQ-5D dimensions of usual activities, pain and anxiety/depression; and had significantly higher utility scores and EQ-VAS scores. Subjects with knee problems reported a significantly lower proportion of limitation in the EQ-5D dimensions of usual activities, pain and anxiety/depression and significantly higher utility scores and EQ-VAS scores when compared with subjects with back pain. The effect sizes of group differences in EQ-5D-5L utility and VAS scores were moderate to large (0.50 to 1.14), indicating high sensitivity.

**Table 4:** Sensitivity of electronic EQ-5D-5L by known group comparison at baseline in 2018 (N = 151)

Group	EQ-5D Dimensions (% with limitation, n) ^#^					EQ-5D (mean ± SD, n)	
	Mobility	Self-care	Usual activities	Pain	Anxiety/depression	Utility score	VAS score
*Pain rating scale *							
Score = 0–5	21.7% (106)	2.8% (106)	9.4% (106)	33.0% (106)	5.7% (106)	0.71 ± 0.19 (106)	71.30 ± 15.98 (106)
Score > 5	44.4% (45)	17.8% (45)	51.1% (45)	82.2% (45)	40.0% (45)	0.43 ± 0.29 (45)	59.84 ± 17.10 (45)
*p* value	0.047	0.001	< 0.001	< 0.001	< 0.001	0.001	< 0.001
Effect size						1.142	0.692
*Clinic setting*							
GOPC	24.7% (101)	6.9% (101)	10.9% (101)	40.6% (101)	9.9% (101)	0.67 ± 0.23 (101)	71.27 ± 15.76 (101)
SOPC	36.0% (50)	8% (50)	44.0% (50)	62% (50)	28% (50)	0.52 ± 0.27 (50)	61.06 ± 17.75 (50)
*p* value	0.074	0.403	< 0.001	0.007	0.002	0.001	< 0.001
Effect size						0.598	0.608
*MS Diagnosis*							
Knee	27.2% (88)	5.7% (88)	1.3% (88)	35.2% (88)	6.8% (88)	0.68 ± 0.22 (88)	71.76 ± 16.75 (88)
Back	30.2% (53)	7.5% (53)	35.8% (53)	67.9% (53)	28.3% (53)	0.55 ± 0.28 (53)	63.60 ± 16.11 (53)
*p* value	0.704	0.674	< 0.001	< 0.001	0.001	0.004	0.005
Effect size						0.516	0.497

MS, musculoskeletal; GOPC ,general outpatient clinic; SOPC, specialist outpatient clinic;

^#^ EQ-5D-5L dimension responses of moderate, severe and very severe were grouped into the ‘with limitation’ category

### Responsiveness

**Fig. 2:**
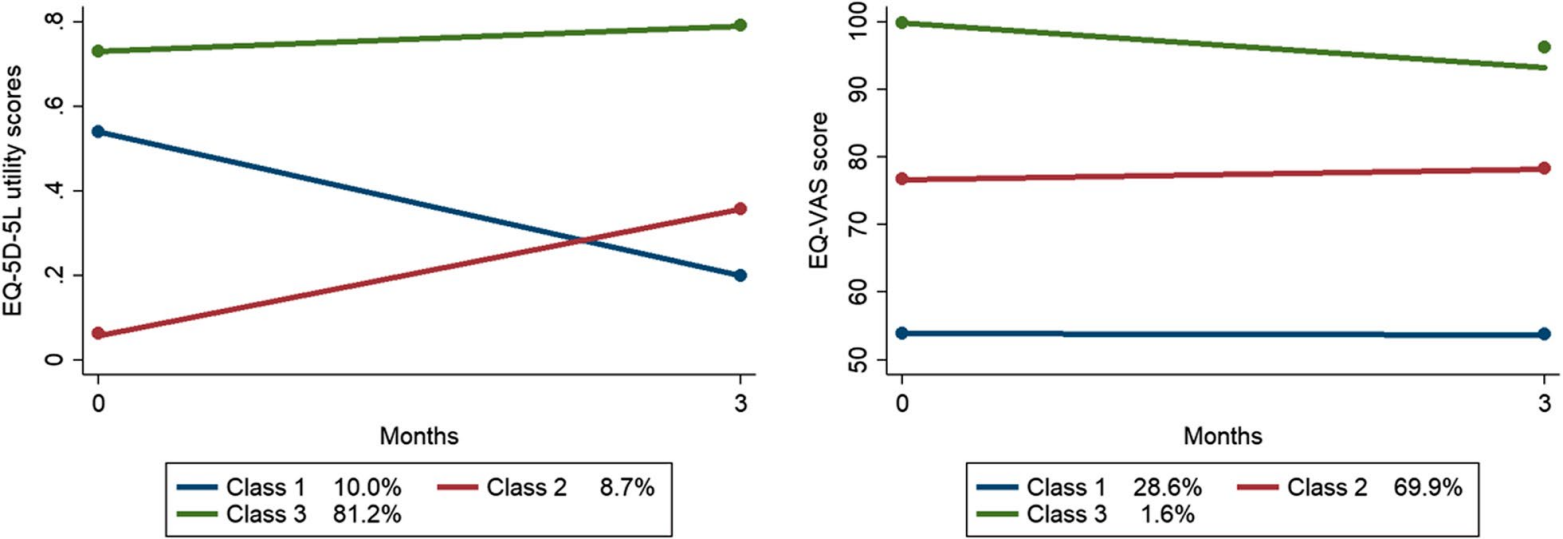
Trajectory analysis

A three-class trajectory model had the best fit for both the longitudinal data of the EQ-5D-5L utility and EQ-VAS scores of 102 subjects according to the BIC. In the EQ-5D-5L utility score model, the class 1 (from middle at baseline to low at follow-up), 2 (from low at baseline to middle at follow-up) and 3 (persistently high) included 10.0%, 8.7%, and 81.2% subjects, respectively. In the EQ-VAS score model, the class 1 (persistently low), 2 (persistently middle) and 3 (persistently high) included 28.6%, 69.9%, and 1.6% subjects, respectively. Plots of the EQ-5D-5L utility and EQ-VAS trajectories are shown in Fig. 2. There were differences in the baseline age, diagnosis and clinic setting among the three EQ-5D Utility classes, with class 3 (persistently high utility) subjects more likely to be younger, diagnosed to have knee problems and attending GOPC. The details are shown in Additional file 2: Tables S1a and S1b.

We evaluated the changes in the EQ-5D-5L utility and EQ-VAS scores and the EQ-5D response level proportions by GRS groups among 99 subjects who had completed both the e-EQ-5D-5L and GRS during their 3-month clinic follow up (Table 5). There were significant increases in mean EQ-5D utility and mean EQ-VAS scores from baseline to three-month follow-up among the GRS ‘better’ group. The effect sizes of change for this group were moderate (utility = 0.666 and VAS = 0.664). There were expected negative changes in both mean EQ-5D-5L Utility score and the VAS score in the GRS ‘worse’ group (effect sizes being 0.280 and 0.296 for utility score and EQ-VAS score, respectively) but the differences did not reach statistical significance. There was also a significant increase in the EQ-5D utility score at 3 months in the GRS “same” group. When looking into the changes in the EQ-5D-5L dimensions, as expected, the GRS ‘better’ group showed a decrease in the proportion of subjects who reported to have limitations/problems across all dimensions whereas an increase was noted amongst subjects who reported ‘worse’ on the GRS. The differences in changes in the proportions of limitations among the GRS groups were statistically significant.

**Table 5:** Change in electronic EQ-5D scores from baseline to 3 months follow up by GRS groups, Aug 2018 – Mar 2019 (N = 99)

	EQ-5D Utility score (mean ± SD, n)				EQ-VAS score (mean ± SD, n)				Change in EQ-5D Dimension level with limitation from baseline to 3-month (%)				
	Baseline	3-month	Difference	*p *value*	Baseline	3-month	Difference	*p *value*	Mobility	Self-care	Usual Activities	Pain/Discomfort	Depression/Anxiety
*GRS Group*													
*GRS Better*	0.58 ± 0.28 (20)	0.76 ± 0.26 (20)	0.18 ± 0.18 (20)	< 0.001	65.90 ± 18.13 (20)	76.65 ± 14.00 (20)	10.75 ± 15.33 (20)	0.005	− 15.0% (20)	− 15.0% (20)	− 15.0% (20)	− 40% (20)	− 20% (20)
Effect size			0.666				0.664						
*GRS Same*	0.71 ± 0.23 (44)	0.79 ± 0.17 (44)	0.08 ± 0.17 (44)	0.002	74.59 ± 15.22 (44)	76.32 ± 11.54 (44)	1.73 ± 12.60 (44)	0.368	− 9.1% (44)	− 2.3% (44)	− 2.3% (44)	− 13.6% (44)	0% (44)
Effect size			0.396				0.128						
*GRS Worse*	0.61 ± 0.27 (35)	0.53 ± 0.30 (35)	− 0.08 ± 0.28 (35)	0.092	67.37 ± 15.22 (35)	62.43 ± 18.08 (35)	− 4.94 ± 18.31 (35)	0.120	14.3% (35)	2.9% (35)	8.6% (35)	22.9% (35)	2.9% (35)
Effect size			0.280				0.296		*p * value#				
									< 0.001	< 0.001	< 0.001	0.021	< 0.001
*Overall*	0.65 ± 0.26 (99)	0.69 ± 0.27 (99)	0.04 ± 0.24 (99)	0.074	70.28 ± 16.33 (99)	71.47 ± 15.97 (99)	1.19 ± 16.24 (99)	0.467	− 2.2% (99)	− 2.0% (99)	− 1.0% (99)	− 6.1% (99)	− 3.0% (99)
Effect size			0.151				0.074						
Mean GRS†			0.589**				0.495**						

GRS, global rating scale

*Difference between baseline and 3-month EQ-5D Utility and EQ-5D VAS scores tested by paired t-tests

^#^EQ-5D-5L dimension responses of moderate, severe and very severe were grouped into the ‘with limitation’ category; difference in proportions among GRS groups tested by Chi-square tests

^†^Correlation between mean GRS score and EQ-5D-5L utility or VAS score, tested by Spearman correlation; **p < 0.001

## Discussion

To the best of our knowledge, this was the first study evaluating the validity and psychometric properties of an electronic version of the EQ-5D-5L in clinical practice in a Chinese population. Our study results demonstrated that the e-EQ-5D-5L was valid, reliable, sensitive and responsive among patients with chronic knee and/or back problems, many of whom were elderly with low education levels. It was reassuring to find that there was no significant difference in EQ-5D-5L scores among subjects with different education levels. The results support the application of the e-EQ-5D-5L in clinical practice, which has the potential to overcome many implementation barriers associated with data collection by paper-based EQ-5D-5L questionnaires, particularly the workload and time to collect, analyze and interpret the data [24]. Another advantage of e-EQ-5D-5L is instantaneous data analysis and generation of a report on the longitudinal data on the HRQOL dimension, utility and VAS outcomes, which can be available at the point of care to support clinical decisions.

As there is no gold standard measure of HRQOL, we could only infer validity of the e-EQ-5D-5L for musculoskeletal problems by comparing the results with those of a musculoskeletal disease specific HRQOL measure, namely WOMAC. The construct validity of the e-EQ-5D-5L was supported by its association with WOMAC scores. The sign of mean differences in WOMAC scores between each adjacent response level in EQ-5D-5L were in the same direction indicating that both measures were measuring the same construct, HRQOL. Due to the small number of respondents (n < 10) in EQ-5D-5L levels 4/5, we grouped the responses of 3/4/5 levels of the EQ-5D-5L dimensions as the ‘3+’ category to increase statistical power for the ANOVA analysis. Validity was further supported by a significant correlation between the EQ-5D Utility and EQ-VAS scores and the WOMAC total score. A study on the validity of the paper version of the EQ-5D-5L among UK patients with rheumatoid arthritis showed similar findings [44], which suggested that the electronic mode of administration did not affect the validity of the EQ-5D-5L in patients with musculoskeletal problems. We noted the difference in the WOMAC scores between subjects who reported level 1 and 2 in the EQ-5D Pain dimension was not significant, but that between levels 1 and 2 of most other EQ-5D dimensions were, which suggest non-linearity either associated with the "gap" between EQ-5D response levels, or to accommodation to pain. The other finding of interest was the Stiffness subscale in WOMAC did not "perform" well against the EQ-5D-5L. One possible explanation is that stiffness was not a significant problem among our subjects who mostly had non-inflammatory knee or back problems. The other explanation is that stiffness may be a subordinate dimension that is indirectly measured through pain/function.

As HRQOL measures are often used to monitor change over time, or with intervention, it is essential for the instrument to have inter-rater/test–retest reliability so that any difference on repeated measurements is a true change in the person’s HRQOL but not measurement variations [29]. Our test–retest ICC results were similar to findings on the paper versions of EQ-5D in Korea [45] and the UK [44]. There was also good test–retest agreement in the results for all five dimensions of the EQ-5D-5L. The findings assure the consistency of the subjects’ responses even when the EQ-5D-5L is presented in an electronic mode that they are not familiar with.

The e-EQ-5D-5L was able to detect significant differences between different known groups, as hypothesized. The effect sizes of the differences in the EQ-5D-5L utility and VAS scores between the known groups were moderate to large (0.50–1.14), suggesting they were likely to be clinically important [46]. It is expected that subjects with mild pain were less likely to report limitations or problems in the EQ-5D-5L dimensions. They also had higher EQ-5D-5L utility scores than those with severe pain. The e-EQ-5D-5L detected lower proportions with HRQOL limitations and higher utility and VAS scores in GOPC than SOPC subjects, which is consistent with the conventional practice that patients with milder problems are managed in primary care. The e-EQ-5D-5L utility and VAS scores showed statistically significant differences between patients with knee and back problems, with moderate effect sizes of 0.52 and 0.50, respectively. A higher proportion of the subjects with back problems were patients from the SOPC who tended to have more severe diseases. Apart from disease severity, other differences in the characteristics of subjects between primary and specialist care clinics could have affected the EQ-5D-5L results, but the small sample in this study did not have the power for clinic-diagnosis subgroup analysis. Further studies should be carried out to identify the other factors associated with HRQOL of chronic knee and/or back patients. The EQ-5D-5L specifically identified significantly more limitations in the pain, usual activities and anxiety/depression but not in mobility or self-care in the patient group with back problems than those with knee problems. The patient’s HRQOL profile can help clinicians identify specific areas of need so that the management can be more tailor-made. In addition to pain relief, strategies to enhance functioning in daily activities and to relieve psychological distress deserve more attention in the care of patients with chronic knee and back problems.

The responsiveness of e-EQ-5D-5L was established in the three-month follow-up measurement. The trend of change was consistent with those measured by the GRS, in that subjects who reported their global health had got better had an increase in EQ-5D-5L utility and EQ-VAS scores and a decrease in the proportions reporting limitations/problems in the EQ-5D-5L dimensions, and vice versa among subjects who reported their global health had got worse. The 3-month changes in the EQ-5D-5L utility and EQ-VAS scores were statistically significant and the effect sizes were moderate (0.666 and 0.664) in the GRS better group. The effect size changes in the EQ-5D-5L utility and EQ-VAS scores were smaller (0.28 to 0.30) in the worsen group and the difference did not reach statistical significance in this small sample. Our findings were consistent with those found in a systematic review by Payakachat et al. in that the EQ-5D was responsive to changes in musculoskeletal and pain conditions more consistently in detecting improvement than deterioration [47]. We noted a statistically significant increase in the EQ-5D-5L utility score in the group who reported the same global health at 3 months. The EQ-VAS score also showed an increase among the GRS same group although the difference was not statistically significant. One possible explanation is that the multi-dimensional EQ-5D-5L is more responsive than a transitional measure on change in global health in detecting a small HRQOL improvement. On the other hand, a small change in the EQ-5D-5L utility score could be “noise” that may not truly reflect a real change. Further studies using different external anchors are required to establish the minimal clinically important change of the EQ-5D-5L.

### Strengths and limitations

To the best of our knowledge, this study was the first to establish the validity and psychometric properties of an electronic version of the EQ-5D-5L as a HRQOL measurement in clinical practice amongst Chinese patients. Knee and back problems are the most common musculoskeletal problems and we included subjects from primary and specialist care who had a broad spectrum of disease severity. We were able to demonstrate the applicability and validity of e-EQ-5D-5L in elderly patients with low education levels who are not as familiar with computer technology. We therefore believe that the e-EQ-5D-5L is likely to be valid in other Chinese patients with musculoskeletal problems.

Our study had some limitations in that the sample size was small, the follow-up period was short (3 months only) and subjects from only two public outpatient clinics were included. We did not use the paper EQ-5D-5L as a ‘gold standard’ criterion to test the validity and concordance of e-EQ-5D-5L, as to do so would require a larger randomized controlled study. Results on validity and psychometric properties do not necessarily imply the e-EQ-5D-5L data are clinically useful. Further studies with longer follow-up period and larger samples from different clinical settings should be carried out to establish the usefulness and acceptability of e-EQ-5D-5L in measuring HRQOL in routine clinical practice. Specifically, we need to determine whether the HRQOL data measured by e-EQ-5D-5L is useful in improving the health outcomes of patients and quality of care. An evaluation on the acceptability to patients and staff, feasibility and resource implication of routine electronic measurement of EQ-5D-5L in clinical practice should also be carried out before implementation.

## Conclusions

Electronic administration of the Chinese (Hong Kong) EQ-5D-5L was found to be valid, reliable, sensitive and responsive for the measurement of HRQOL of Chinese patients with chronic knee and/or back problems in routine clinical practice. We are now ready to proceed to the next research study to determine the clinical usefulness of the e-EQ-5D-5L data in improving health outcomes. If proven to be useful, the e-EQ-5D-5L can be incorporated into electronic medical record systems to facilitate the evaluation and monitoring of HRQOL as part of routine clinical care for patients with chronic musculoskeletal problems.

## Supplementary Information

**Additional file 1: Appendix 1**. Electronic EQ-5D-5L and EQ-VAS Completion Procedure with Screenshots

**Additional file 2: Table S1a**. Baseline characteristics of subjects by three trajectory classes of EQ-5D-5L utility scores. **Table S1b**. Baseline characteristics of subjects by three trajectory classes of EQ-VAS scores

## Data Availability

The datasets used and/or analyzed during the current study are available from the corresponding author on reasonable request.

## Abbreviations

EQ-5D-5L: EuroQol-5 dimensions-5 levels
HRQOL: Health-related quality of life
WOMAC: Western Ontario and McMaster University Osteoarthritis Index
ANOVA: Analysis of variance
VAS: Visual analogue scale
ICC: Intraclass correlation coefficient
EQ-5D-3L: EuroQol-5 dimensions-3 levels
GOPC: General out-patient clinic
GRS: Global Rating Scale
IBM: International Business Machines Corporation
SPSS: Statistical Product and Service Solutions
AC: Agreement coefficients
SOPC: Specialist out-patient clinic
SD: Standard deviations
