# Supplementary material for: A pilot study on the validity and psychometric properties of the electronic EQ-5D-5L in routine clinical practice
Source: Health Qual Life Outcomes. 2021 Dec 18;19:266. doi: 10.1186/s12955-021-01898-3 (PMC8684117; doi:10.1186/s12955-021-01898-3)
Supplement: Supplementary file 1 — Additional file 1: Appendix 1. Electronic EQ-5D-5L and EQ-VAS Completion Procedure with Screenshots [file 12955_2021_1898_MOESM1_ESM.pdf]

Supplementary material: Appendix 1

Electronic EQ-5D-5L and EQ-VAS Completion Procedure with Screenshots

<table>
<tr><td colspan="2">Step 1 – Each subject is assigned a unique subject number with QR code to log into the system to complete the EQ-5D-5L and EQ-VAS online</td></tr>
<tr><td>Screenshot 1</td><td>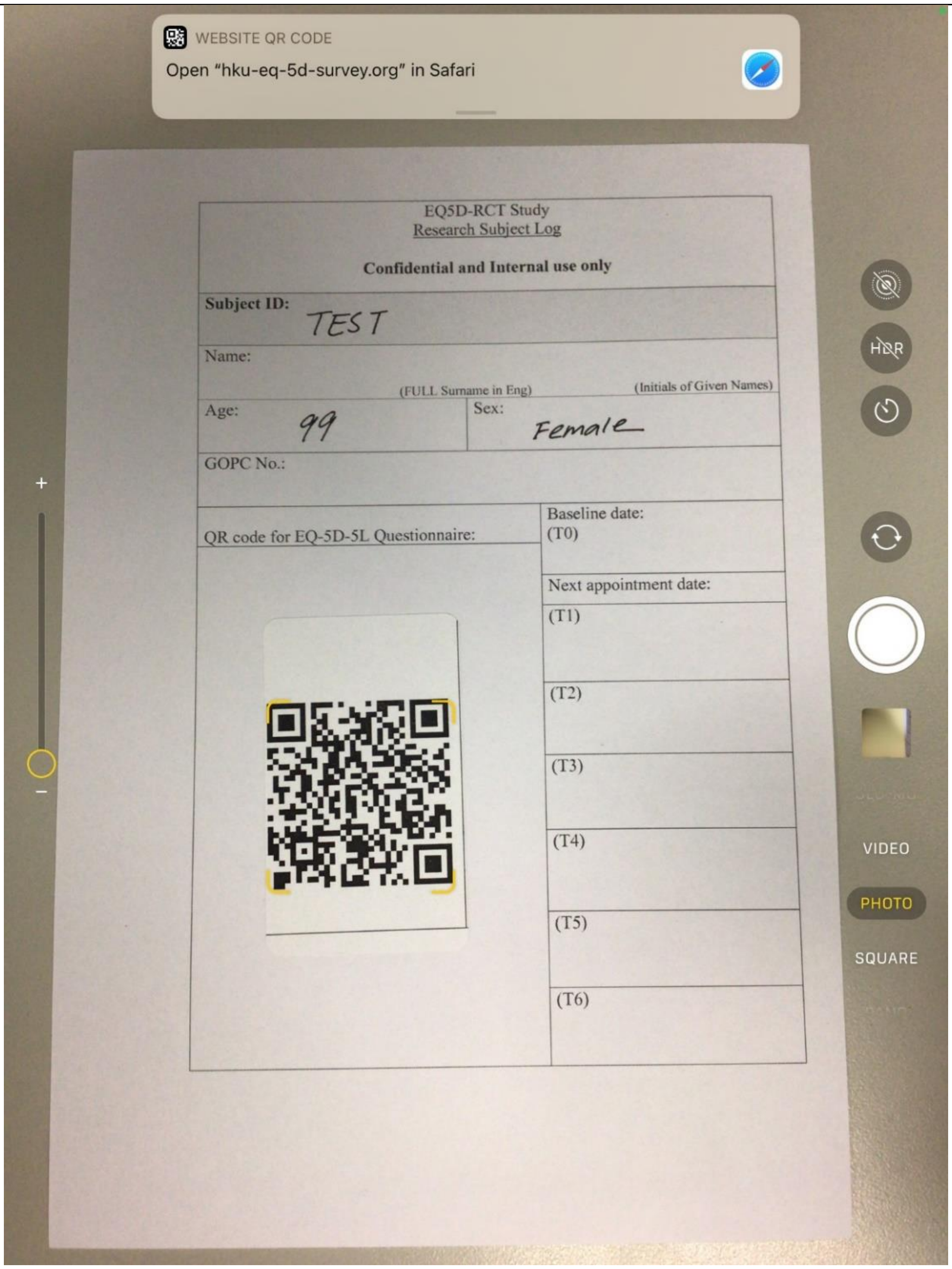
</td></tr>
</table>

Step 2 – Enter the age and gender for linkage to the relevant Hong Kong age-gender specific population norm reference on the report

Screenshot 2

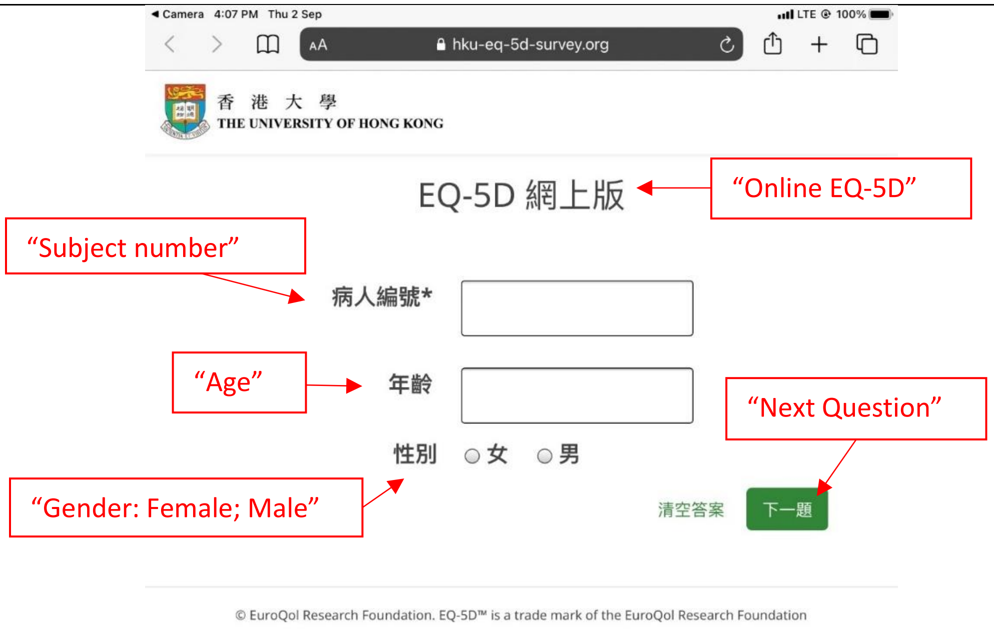

**Step 3 – Completion of the EQ-5D item on Mobility**<br>**The instruction is shown before the item and the response options.**	
**Screenshot 3**	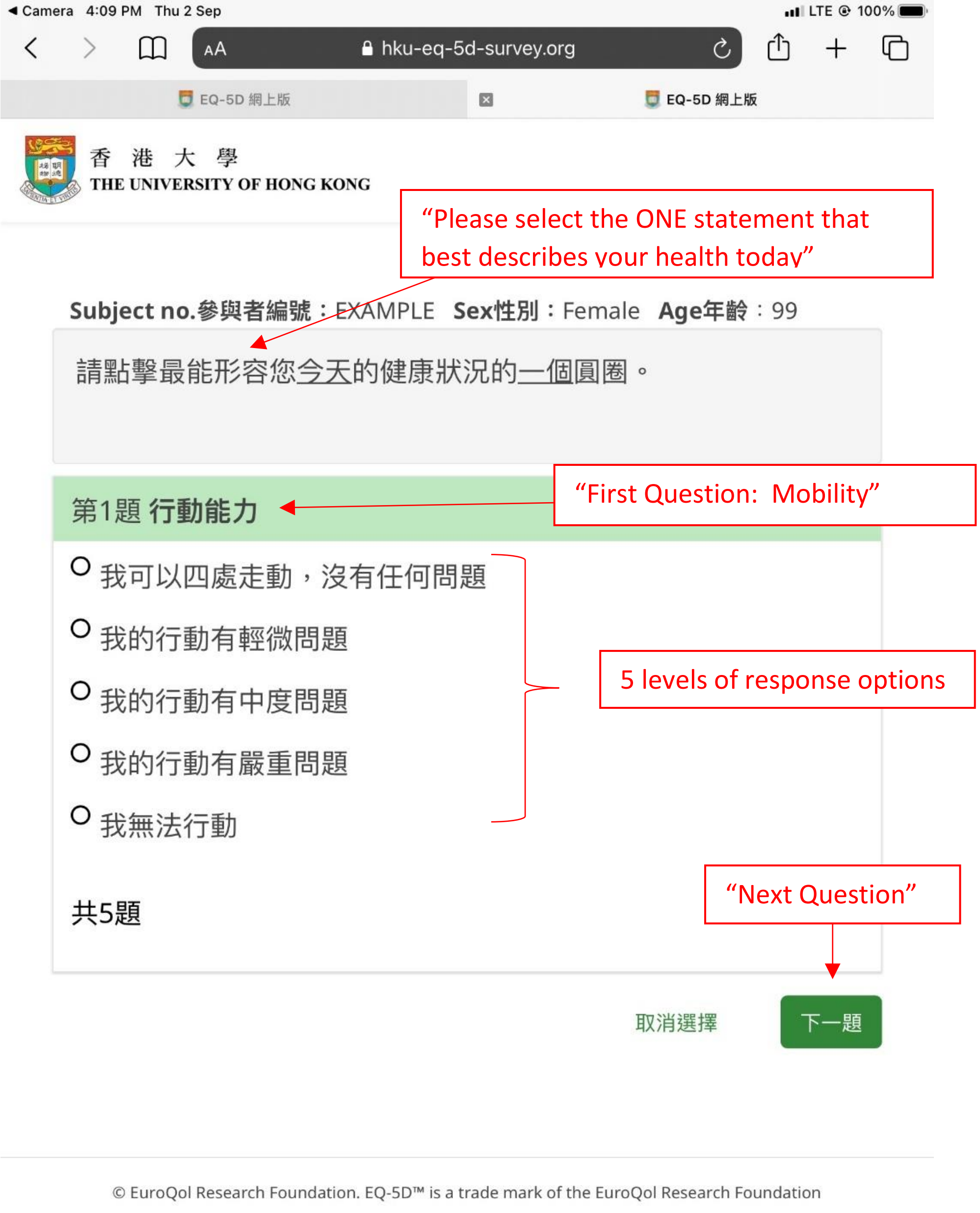
**N.B.**	The subject needed to click "Next" before moving to the next item. The subject could choose not to provide an answer (i.e. skip the question) before moving to the next.

**Step 4-7 – Completion of EQ-5D items on Self-care, Daily Activities, Pain and Emotion, each presented on one screen at a time with instruction shown before the item.**	
**Screenshot 4-7**	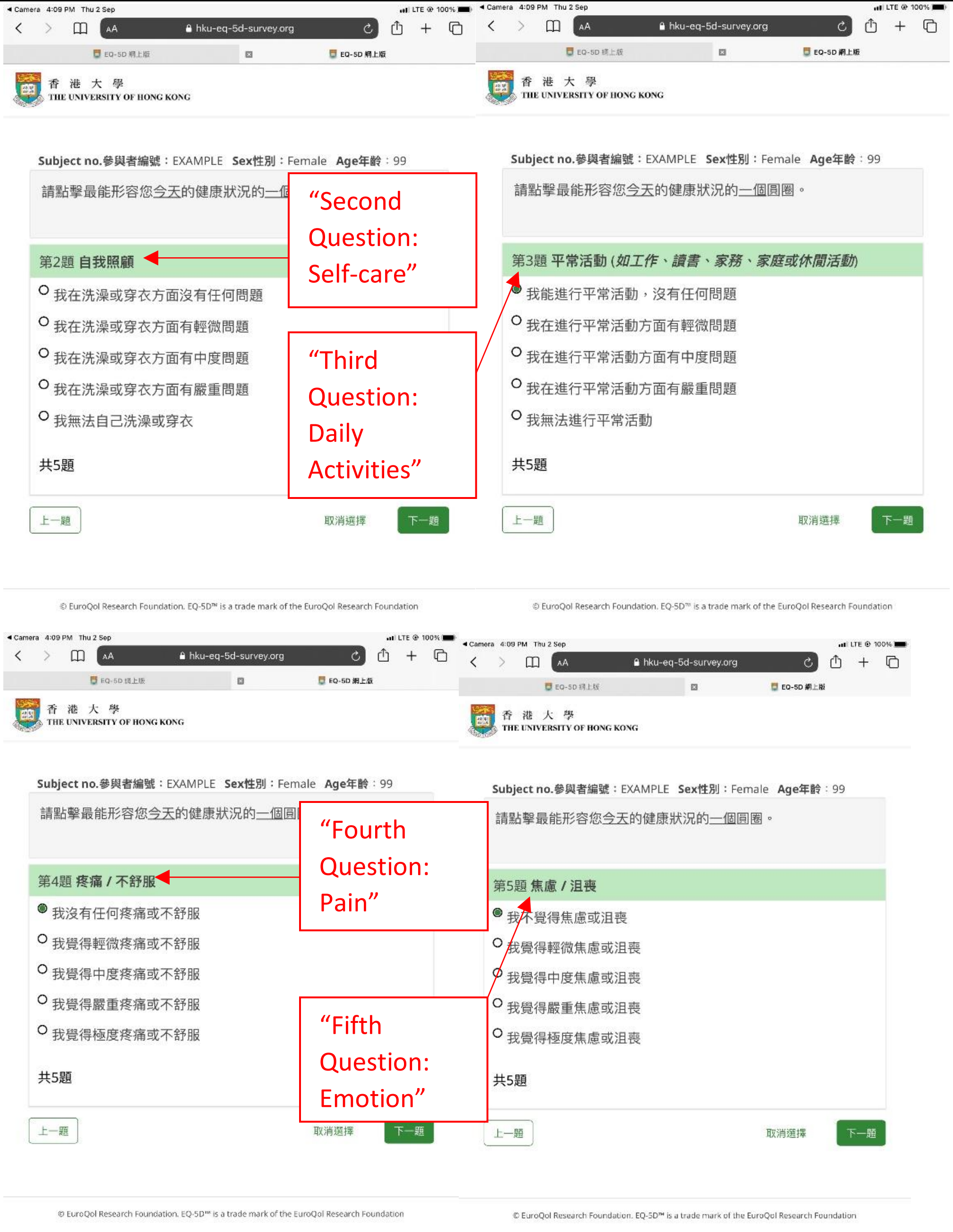
**N.B.**	The subject needed to click “Next” before moving to the next item. The subject could choose not to provide an answer (i.e. skip the question) before moving to the next

<table>
<tr><td colspan="2">Step 8 – Completion of the EQ-VAS, the instruction is shown on the left and the subject needed to enter the appropriate number (0 to 100) in the box.</td></tr>
<tr><td>Screenshot 8</td><td>
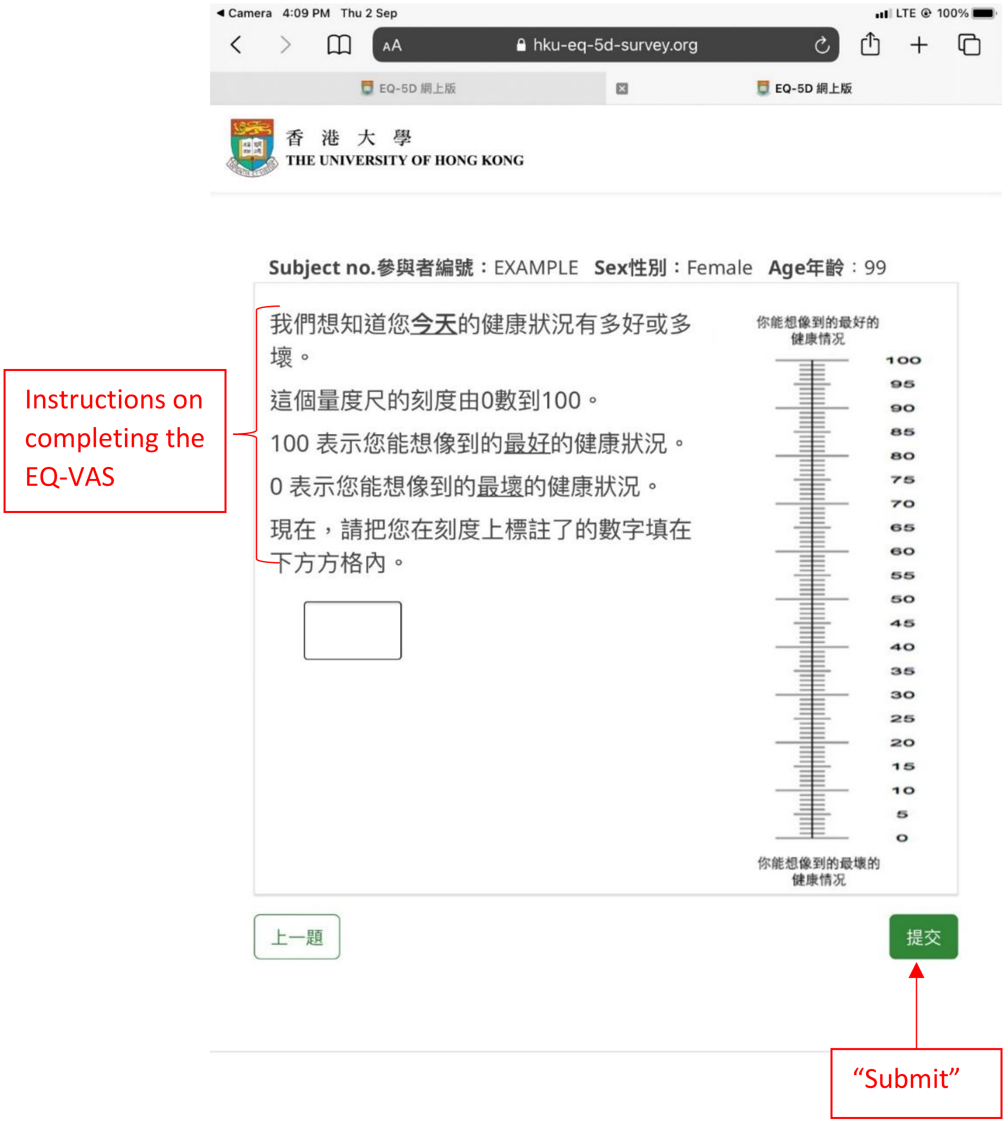

</td></tr>
<tr><td>N.B.</td><td>the EQ-VAS was modified from the original 200mm to a 100mm scale from 0 (the worst imaginable health state) to 100 (the best imaginable health state), in order to fit into one page of the screen. To proceed, subjects needed to click “Submit” after this item.</td></tr>
</table>

**Step 9 - Thank you – Your answers have been recorded**	
**Screenshot 9**	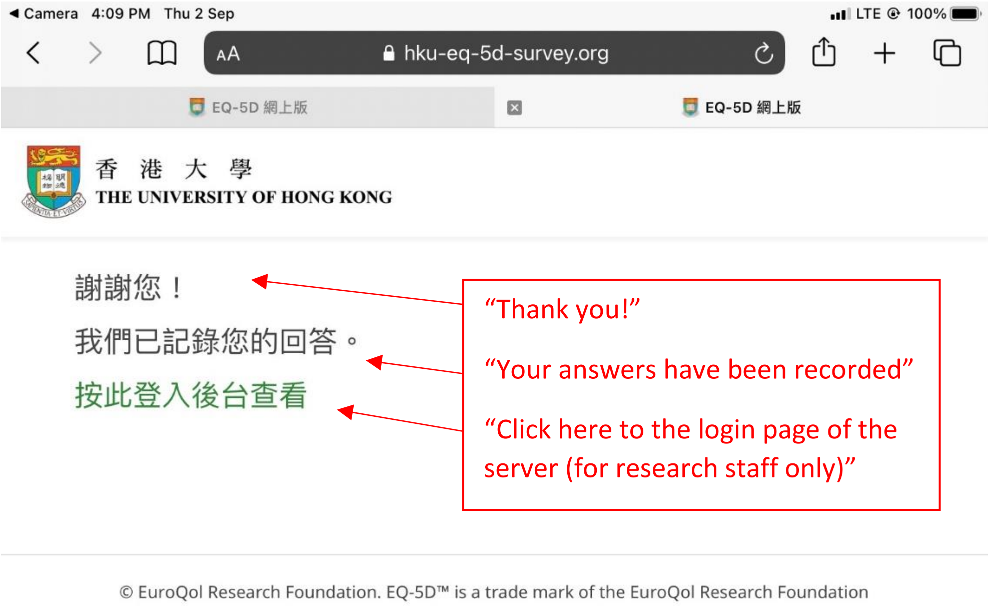
**N.B.**	After the subject has completed the electronic EQ-5D-5L and EQ-VAS, a report on the EQ-5D-5L, utility and VAS scores would be generated within one minute.

**Step 10 – Trained Research Assistant would Login the Sever to Retrieve the EQ-5D Report**	
**Screenshot 10**	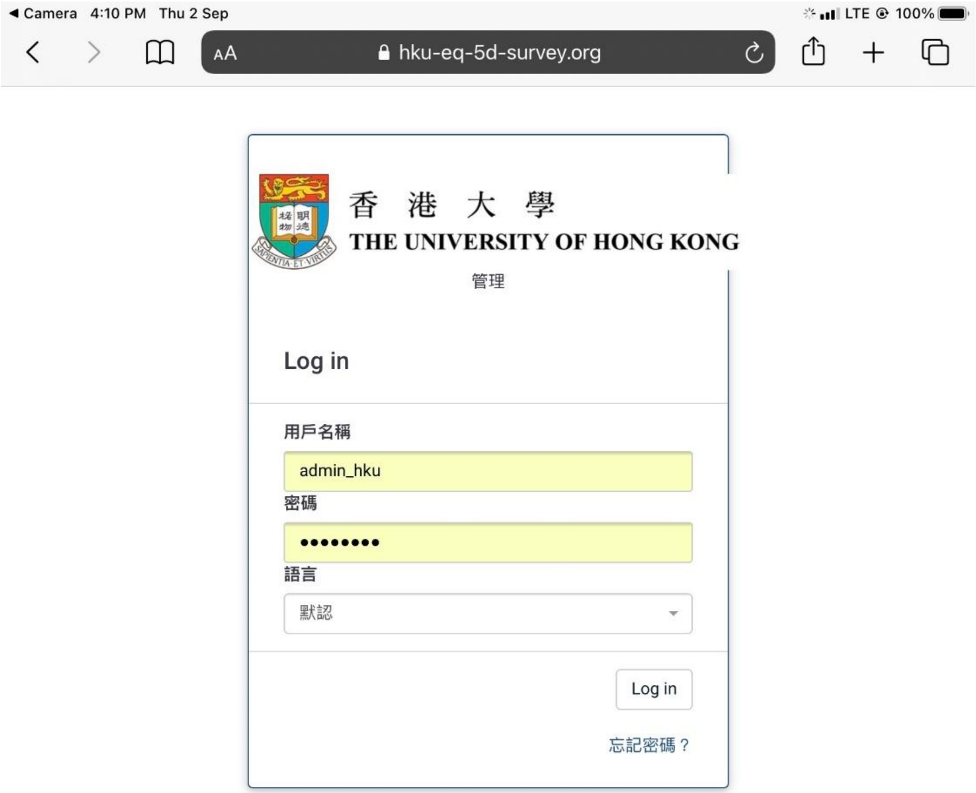
**N.B.**	To ensure data privacy and protection, credentials and password, which are only available to the study team, were required to log into the server

Step 11 – Printable EQ5D report

Screenshot 11

12:54 PM Mon 6 Sep 66%

hku-eq-5d-survey.org

1 of 1

Subject No. 參與者編號: 003 Sex性別: Female Age年齡: 51

EQ-5D-5L Level: 1=no problem無問題, 2=slight problems有輕微問題, 3=moderate problems有中度問題, 4=severe problems有嚴重問題, 5=extreme problems有極度問題

	HK population median	2018 08-13 10:08	2018 11-12 09:19
Mobility 行動能力	1	2	1
Self-Care 自我照顧	1	1	1
Usual activities 平常活動	1	1	1
Pain/discomfort 疼痛/不舒服	1	3	1
Anxiety/depression 焦慮/沮喪	2	2	1

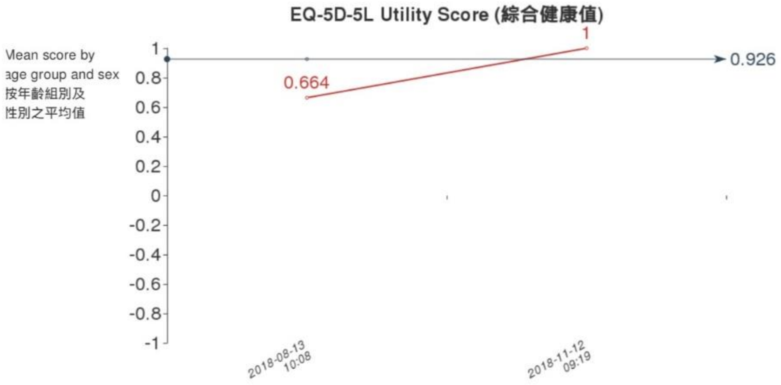

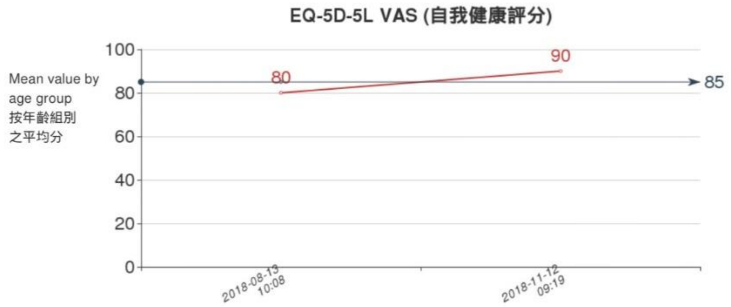

N.B.

The report included the subject's longitudinal EQ-5D dimension, utility and VAS scores, up to five records, and the Hong Kong age-gender specific population norm reference.
