# Supplementary material for: A pilot study on the validity and psychometric properties of the electronic EQ-5D-5L in routine clinical practice
Source: Health Qual Life Outcomes. 2021 Dec 18;19:266. doi: 10.1186/s12955-021-01898-3 (PMC8684117; doi:10.1186/s12955-021-01898-3)
Supplement: Supplementary file 2 — Additional file 2: Table S1a. Baseline characteristics of subjects by three trajectory classes of EQ-5D-5L utility scores. Table S1b. Baseline characteristics of subjects by three trajectory classes of EQ-VAS scores [file 12955_2021_1898_MOESM2_ESM.pdf]

Supplementary Table 1a. Baseline characteristics of subjects by three trajectory classes of EQ-5D-5L utility scores

Baseline characteristics, % (n) / mean (SD)	Class 1 (N=9)	Class 2 (N=10)	Class 3 (N=83)	P-value	P-value 1 vs. 2	P-value 1 vs. 3	P-value 2 vs. 3
Gender				0.976	0.845	0.951	0.839
Male	55.6% (5)	60.0% (6)	56.6% (47)				
Female	44.4% (4)	40.0% (4)	43.4% (36)				
Age, year	66.4 (7.6)	71.9 (11.0)	65.2 (9.2)	0.103	0.232	0.703	0.036**
Diagnosis				0.047*	0.756	0.022**	0.159
Back	55.6% (5)	40.0% (4)	21.7% (18)				
Knee	33.3% (3)	50.0% (5)	75.9% (63)				
Both	11.1% (1)	10.0% (1)	2.4% (2)				
Outpatient clinic				0.004*	0.845	0.006**	0.012**
General	55.6% (5)	60.0% (6)	89.2% (74)				
Specialist	44.4% (4)	40.0% (4)	10.8% (9)				
Education level				0.258	0.408	0.714	0.102
None	11.1% (1)	40.0% (4)	12.1% (10)				
Primary or below	44.4% (4)	30.0% (3)	28.9% (24)				
Secondary	33.3% (3)	30.0% (3)	51.8% (43)				
Tertiary level or above	11.1% (1)	0.0% (0)	7.2% (6)				
EQ-5D utility score	0.55 (0.13)	0.07 (0.19)	0.73 (0.16)	<0.001*	<0.001**	0.001**	<0.001**
EQ-VAS score	64.4 (15.9)	50.8 (17.3)	73.7 (14.4)	<0.001*	0.093	0.073	<0.001**

* Significant at 0.05 level by one-way analysis of variance or chi-square test, as appropriate.

** Significant at 0.05 level by independent t-test or chi-square test, as appropriate.

Supplementary Table 1b. Baseline characteristics of subjects by three trajectory classes of EQ-VAS scores

Baseline characteristics, % (n) / mean (SD)	Class 1 (N=28)	Class 2 (N=72)	Class 3 (N=2)	P-value	P-value 1 vs. 2	P-value 1 vs. 3	P-value 2 vs. 3
Gender				0.387	0.554	0.265	0.198
Male	60.7% (17)	54.2% (39)	100.0% (2)				
Female	39.3% (11)	45.8% (33)	0.0% (0)				
Age, year	68.7 (8.9)	64.9 (9.6)	66.0 (1.4)	0.204	0.077	0.677	0.877
Diagnosis				0.619	0.421	0.538	0.683
Back	32.1% (9)	25.0% (18)	0.0% (0)				
Knee	60.7% (17)	72.2% (52)	100.0% (2)				
Both	7.1% (2)	2.8% (2)	0.0% (0)				
Outpatient clinic				0.333	0.184	0.419	0.571
General	75.0% (21)	86.1% (62)	100.0% (2)				
Specialist	25.0% (7)	13.9% (10)	0.0% (0)				
Education level				0.907	0.702	0.798	0.900
None	21.4% (6)	12.5% (9)	0.0% (0)				
Primary or below	25.0% (7)	31.9% (23)	50.0% (1)				
Secondary	46.4% (13)	48.6% (35)	50.0% (1)				
Tertiary level or above	7.1% (2)	6.9% (5)	0.0% (0)				
EQ-5D utility score	0.49 (0.30)	0.71 (0.21)	1.00 (0.00)	<0.001*	<0.001**	0.025**	0.051
EQ-VAS score	53.0 (15.1)	76.7 (10.2)	100.0 (0.0)	<0.001*	<0.001**	<0.001**	0.002**

* Significant at 0.05 level by one-way analysis of variance or chi-square test, as appropriate.

** Significant at 0.05 level by independent t-test or chi-square test, as appropriate.
